# A standardized nomenclature and atlas of the female terminalia of *Drosophila melanogaster*

**DOI:** 10.1080/19336934.2022.2058309

**Published:** 2022-05-14

**Authors:** Eden W. McQueen, Mehrnaz Afkhami, Joel Atallah, John M. Belote, Nicolas Gompel, Yael Heifetz, Yoshitaka Kamimura, Shani C. Kornhauser, John P. Masly, Patrick O’Grady, Julianne Peláez, Mark Rebeiz, Gavin Rice, Ernesto Sánchez-Herrero, Maria Daniela Santos Nunes, Augusto Santos Rampasso, Sandra L. Schnakenberg, Mark L. Siegal, Aya Takahashi, Kentaro M. Tanaka, Natascha Turetzek, Einat Zelinger, Virginie Courtier-Orgogozo, Masanori J. Toda, Mariana F. Wolfner, Amir Yassin

**Affiliations:** a Department of Biological Sciences, University of Pittsburgh, Pittsburgh, PA, USA; b Department of Molecular, Cellular, and Developmental Biology, University of Michigan, Ann Arbor, MI, USA; c Department of Biology, University of Oklahoma, Norman, OK, USA; d Department of Biological Sciences, University of New Orleans, New Orleans, LA, USA; e Department of Biology, Syracuse University, Syracuse, NY, USA; f Evolutionary Ecology, Ludwig-Maximilians Universität München, Fakultät für Biologie, Biozentrum, Planegg-Martinsried, Germany; g Department of Entomology, The Hebrew University of Jerusalem, Rehovot, Israel; h Department of Biology, Keio University, Yokohama, Japan; i Biozentrum, University of Basel, Basel, Switzerland; j Department of Entomology, Cornell University, Ithaca, NY, USA; k Department of Integrative Biology, University of California, Berkeley, CA, USA; l Centro de Biología Molecular Severo Ochoa (C.S.I.C.-U.A.M.), Universidad Autónoma de Madrid, Cantoblanco, Spain; m Department of Biological and Medical Sciences, Oxford Brookes University, Oxford, UK; n Center for Genomics and Systems Biology, Department of Biology, New York University, New York, NY, USA; o Sema4, Stamford, CT, USA; p Department of Biological Sciences, Tokyo Metropolitan University, Hachioji, Japan; q Research Center for Genomics and Bioinformatics, Tokyo Metropolitan University, Hachioji, Japan; r Center for Scientific Imaging, The Hebrew University of Jerusalem, Rehovot, Israel; s CNRS UMR7592, Institut Jacques Monod, Université Paris Cité, Paris, France; t Hokkaido University Museum, Hokkaido University, Sapporo, Japan; u Department of Molecular Biology and Genetics, Cornell University, Ithaca, NY, USA; v Laboratoire Evolution, Génomes, Comportement, Ecologie (EGCE), UMR 9191, CNRS, IRD, Université Paris-Saclay, Gif-sur-Yvette Cedex, France

**Keywords:** Genitalia, terminalia, anatomy, *Drosophila melanogaster*, nomenclature

## Abstract

The model organism *Drosophila melanogaster* has become a focal system for investigations of rapidly evolving genital morphology as well as the development and functions of insect reproductive structures. To follow up on a previous paper outlining unifying terminology for the structures of the male terminalia in this species, we offer here a detailed description of the female terminalia of *D. melanogaster*. Informative diagrams and micrographs are presented to provide a comprehensive overview of the external and internal reproductive structures of females. We propose a collection of terms and definitions to standardize the terminology associated with the female terminalia in *D. melanogaster* and we provide a correspondence table with the terms previously used. Unifying terminology for both males and females in this species will help to facilitate communication between various disciplines, as well as aid in synthesizing research across publications within a discipline that has historically focused principally on male features. Our efforts to refine and standardize the terminology should expand the utility of this important model system for addressing questions related to the development and evolution of animal genitalia, and morphology in general.

## Introduction

Animal terminalia (which includes both the genitalia and analia) have a long history of being used for taxonomic and phylogenetic purposes, as well as being studied in the context of functional morphology and morphological evolution. This is because these structures possess a remarkable level of anatomical diversity, making them excellent morphological features for distinguishing species as well as understanding mechanisms of rapid morphological change [1–3]. Past investigations mostly focused on male terminalia, and female terminalia were generally considered to be relatively invariable [1,4]. In the last several decades however, there has been a burgeoning interest in improving our understanding of female genital diversity [5–11]. This interest has been motivated by the realization that some evolutionary hypotheses, for instance with respect to coevolution of genitalia, are best addressed by studying both male and female genital morphology simultaneously [3,11–15]. In addition, the female terminalia can evolve in response to ecological factors, such as the properties of egg-laying substrates [6]. Furthermore, as morphological adaptations in female genitalia are central to the ability of many pest species to damage crops when laying their eggs into plants [16–20], studying female genitalia can potentially lead to practical applications.

In recent years, the genitalia of species in the *Drosophila* genus have become an important study system to address research questions in ecology, behaviour, evolution, development and taxonomy. A survey of the egg-laying apparatus of Hawaiian drosophilids for example revealed that ovipositor form, and especially length and patterns of sensory structures, differ between species and strongly correlate with adaptations to different oviposition substrates [6]. Similar observations were made for *Drosophila suzukii*, which has evolved an elongated ovipositor with derived sensory structures, enabling piercing through the skin of still-ripening fruits, which allows this species to access a new ecological niche and simultaneously makes it a pest causing massive agricultural damage [8,16].

Cross-disciplinary communication among researchers investigating different aspects of *Drosophila* female terminalia has often been impeded by two important challenges. First, many important features are internal, mostly composed of folded soft tissues, which can make it more difficult to identify, delimit and rigorously quantify variation in shape between individuals or species. For example, commonly used terms such as vulva, vagina and uterus have no clear delineations and have been applied to variable portions of the genitalia in different publications (see Table 1). Imaging and dissecting technology developed in recent years has greatly mitigated this technical limitation [5]. For instance, micro-computed tomography (micro CT) scanning can now provide detailed images of internal structures [21]. The second challenge has been that individual structures have often been referred to by several different names. This is most obvious in the long list of synonyms that have been applied to the egg-laying sclerites laterally surrounding the gonopore (e.g. ovipositor, vaginal plates, oviscapt, gonopod, etc. See Table 1).

**Table 1.** Definition of the terms in the standardized nomenclature

**Figure 1.**
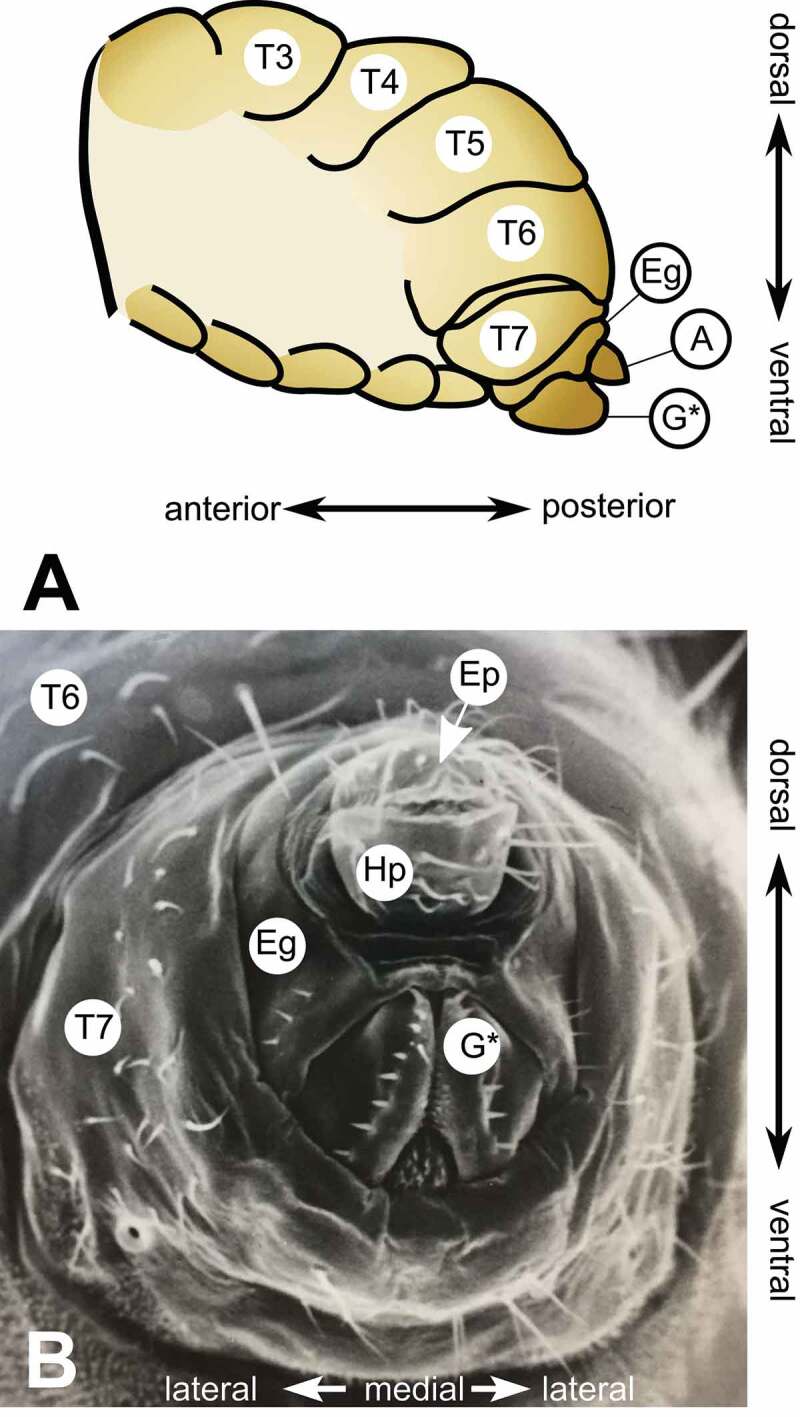
**The terminalia of female *D. melanogaster*. [A]** Model diagram of posterior female abdomen of *D. melanogaster*, lateral view. **[B]** Scanning electron micrograph of *D. melanogaster* female terminalia, posterior view. **T3-T7** = female abdominal tergites 3–7. G* = female genitalia, **A** = female analia, **Eg** = epigynium (T8), **Hp** = hypoproct, **Ep** = epiproct. The hypoproct and the epiproct together form the female analia. *Note that the female genitalia includes the epigynium, which is indicated separately in this figure.

**Figure 2.**
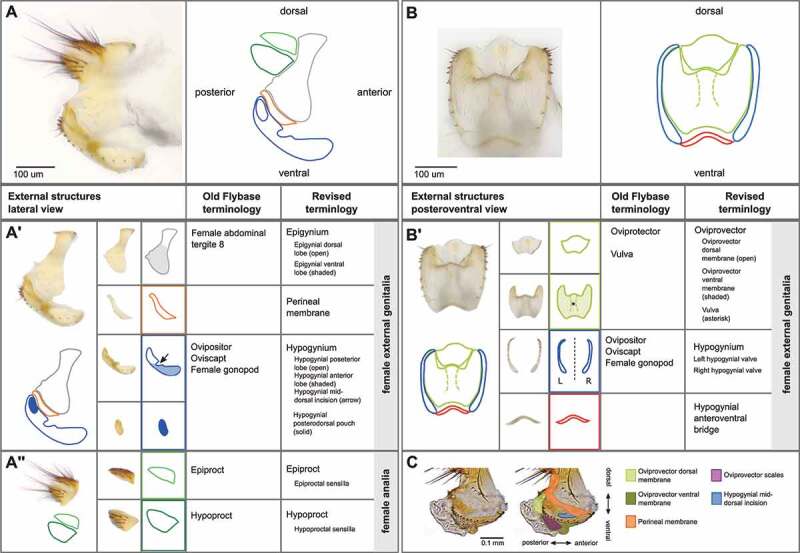
**Visual atlas of the external female terminalia**. Light microscopy images showing the whole external terminalia in lateral view (**panel A**) and the genitalia in posteroventral view (**panel B**). Individual structures are highlighted below each image, with line drawings to aid identification. Previous FlyBase terms are listed in the left column and revised terms are given in the right column. **Panel C** is a detail of a lateral view with internal structures extruded (as during egg laying), to highlight interior membranous structures.

**Parts**
**Female terminalia**
Flybase ID: FBbt:00004830
Definition: The entire set of internal and external structures in the distal half of the female abdomen that are derived from segments 8-10, making up the *female genitalia* and *female analia*. It develops from the female genital disc [24,25].
**Female genitalia**
Flybase ID: FBbt:00004827
Definition: Set of internal and external structures originating from segments 8-9, that makes up the genital apparatus. It develops from the female genital-primordium part of the genital disc ,^25^].
**Female analia**
Flybase ID: FBbt:00004824
Definition: Set of external structures originating from segment 10, that makes up the anal apparatus. It develops from the female genital disc [24,25].
Synonyms: female proctiger [31,42].
**Sclerites**
**Epigynium**
FlyBase ID: FBbt:00110704
FlyBase synonyms: female abdominal tergite 8
Definition: Horseshoe-shaped tergite which, dorsally, surrounds the female analia. It is chitinized, and each side (left and right) is divided into the epigynial dorsal lobe and the epigynial ventral lobe.
Synonyms: female abdominal tergite 8 [24,42,45,48,68–70], female abdominal tergite 9 [31,71].
**Epigynial dorsal lobe**
FlyBase ID: FBbt:00052057
FlyBase synonyms: New term
Definition: Dorsal portion of the epigynium above the epigynial ventral lobe. The two dorsal lobes (left and right) are fused dorsally into a single sclerite. It does not normally harbor sensilla (bristles).
Synonyms: dorsal part of the female abdominal tergite 8 [69].
**Epigynial ventral lobe**
FlyBase ID: FBbt:00052058
FlyBase synonyms: New term
Definition: Ventral portion of the epigynium below the epigynial dorsal lobe. There are two of these, one on each side. Each lobe (left and right) normally harbors four or five small, unpigmented sensilla (bristles).
Synonyms: ventral part of the female abdominal tergite 8 [69].
**Epiproct**
FlyBase ID: FBbt:00004833
Definition: The plate dorsally surrounding the anus in females. It has an average of 18 sensilla of which two are large. It arises from segment 10 primordium in the female genital disc [30,45,46,72,48,49].
Synonyms: abdominal tergite 9 [42], abdominal tergite 10 [56], dorsal anal plate [24,68,70,73–75], upper anal plate [40], supraanal plate [76].
**Female accessory gland**
FlyBase ID: FBbt:00004914
Definition: Small, bilaterally paired gland that lies caudal to the spermathecae and is connected to the uterus by a duct. The gland wall consists of a single layer of secretory cells, each with a large vacuole and a minute acidophilic granule towards the gland lumen. It arises from segment 9 primordium in the female genital disc.
Synonyms: appendicular gland [56], colleterial gland [56], parovarium [24,56,73,77–80].
**Female accessory gland duct**
FlyBase ID: FBbt:00004915
Definition: A duct connecting the female accessory gland to the uterus, opening just caudal to the spermathecal ducts. It consists of a tube of thin epithelium lined with a thin chitinous intima, irregularly ringed with sharp ridges that project into the lumen.
**Female accessory gland fat body**
FlyBase ID: FBbt:00052059 (female accessory gland fat mass)
FlyBase synonyms: New term
Definition: Adipose tissue surrounding the female accessory gland. This tissue may be attached to the fat body surrounding the spermatheca. The female accessory gland fat body is in close contact with the rectum [81].
**Furca**
FlyBase ID: FBbt:00052060 (genital chamber furca)
FlyBase synonyms: New term
Definition: The dorsal wall of the genital chamber that developmentally arises from segment 9 primordium in the female genital disc. It extends from the female accessory gland ducts anteriorly to the vulva posteriorly (Figures 6). It is divided into a uterine furca, in which the female accessory gland duct open and which has an inner posterior thickness called papillate elevation, and a vaginal furca which extrudes from the vulva during oviposition.
Synonyms: female sternite 9 [56], genital fork [56], vaginal apodeme [56].
**Genital chamber**
FlyBase ID: FBbt:00004925
FlyBase synonyms: Revised term. Previously used synonyms include vagina (now FBbt:00052061) and uterus (FBbt:00004924).
Definition: An elongate muscular pouch, the anterior part of which is the uterus, where eggs are fertilized, and the posterior part of which is the vagina, where insemination takes place during copulation. It opens posteriorly through the vulva.
Synonyms: uterus [39,73,79].
**Hypogynium**
FlyBase ID: FBbt:00004832
FlyBase synonyms: female gonopod
Definition: Female abdominal sternite 8 modified for oviposition. It consists of paired chitinous valves (left and right) that are anteriorly connected through the hypogynial anteroventral bridge and posteriorly surrounds the vulva. The ventral margin of each valve is strongly sclerotized and contiguous with the hypogynial anteroventral bridge. Each valve carries 11-16 marginal and apical teeth on its outer wall and four trichoid apical sensilla on its inner wall, the ventral most of which is the hypogynial long sensillum. The dorsal margin of each valve harbors a mid-dorsal incision. An imaginary line connecting the hypogynial long sensillum to the hypogynial mid-dorsal incision delimits the borders between the posterior and anterior lobes of each valve. The posterior lobe of each valve carries a posterodorsal pouch. The anterior lobes of the hypogynial valves are internally connected by the oviprovector. [82,83]
Synonyms: egg-guide [77,80], female gonopod [42], female abdominal sternite 8 [56], ovipositor [35,40,48,75], oviscape [30,31,47,84], oviscapt [15,30,45–47,72,49,85,86], vaginal plates [24,39,68,73,74,79,87].
**Hypogynial anterior lobe**
FlyBase ID: FBbt:00052064
FlyBase synonyms: New term
Definition: The anterior portion of each hypogynial valve, anterior to an imaginary line connecting the hypogynial long sensillum to the hypogynial mid-dorsal incision. It is double-walled, slightly rounded and carries a series of teeth that are smaller and more interspaced than those carried by the hypogynial posterior lobe.
Synonyms: lower margin of the egg-guide lobe [80], ventral vaginal plate [69,70,74,88].
**Hypogynial anteroventral bridge**
FlyBase ID: FBbt:00052062
FlyBase synonyms: New term
Definition: A transverse, strongly sclerotized rod connecting the anterior tips of the hypogynial valves beneath the oviprovector and the vagina.
Synonyms: basal isthmus [77,80], heavily sclerotized bar [42].
**Hypogynial mid-dorsal incision**
FlyBase ID: FBbt:00052065
FlyBase synonyms: New term
Definition: A mid-dorsal incision on the dorsal margin of each hypogynial valve.
Synonyms: submedian incision of the egg-guide lobe [80].
**Hypogynial posterior lobe**
FlyBase ID: FBbt:00052063
FlyBase synonyms: New term
Definition: The posterior portion of each hypogynial valve, posterior to an imaginary line connecting the hypogynial long sensillum and the hypogynial mid-dorsal incision. It is double-walled and carries on the outer wall a series of sensilla that are larger and less interspaced than those carried by the anterior lobe. On the inner wall, there are three terminal, minute sensilla trichoidea and one subterminal, long sensillum. The outer wall is sclerotized, lobate, and harbors the posterodorsal pouch.
Synonyms: upper margin of the egg-guide lobe [80], mesal surface of the oviscapt [72], distal part of the oviscapt valve [46], dorsal vaginal plate [69,70,74,88].
**Hypogynial posterodorsal pouch**
FlyBase ID: FBbt:00052066
FlyBase synonyms: New term
Definition: A posterodorsal depression on the outer wall of the hypogynial posterior lobe of each hypogynial valve.
Synonyms: dorsodistal depression [80], oviscapt pouch [15].
**Hypoproct**
FlyBase ID: FBbt:00004834
Definition: The plate ventrally surrounding the anus in females [30,45,46,72,48,49]. It has an average of 19 sensilla of which four are large. It arises from segment 10 primordium in the female genital disc.
Synonyms: abdominal sternite 9 [42], subanal plate [76], ventral anal plate [24, 68, 70, 73, 74; 75], lower anal plate [40].
**Oviduct**
FlyBase ID: FBbt:00004911
Definition: Duct of the female reproductive tract that connects the ovaries to the uterus [24,39,73]. Oviducts are divided into two lateral oviducts (each connected to an ovary) and one common oviduct, to which the lateral oviducts connect, and which itself connects to the uterus.
**Oviduct, calyx of**
FlyBase ID: FBbt:00004918 (calyx of oviduct)
FlyBase synonyms: New term
Definition: The anterior-most, cup-shaped region of the lateral oviduct [64,89]. Formed by the joining together of the individual pedicels (small tubes coming from the base of each ovariole).
**Oviduct, common**
FlyBase ID: FBbt:00004913 (common oviduct)
Definition: Epithelial tube that connects the lateral oviducts to the uterus [34,64,90–94]. It is lined with a chitinous intima, and surrounded by circular muscles.
**Oviduct dorsal ridge**
FlyBase ID: FBbt:00052068
FlyBase synonyms: New term
Definition: A ridge in the oviduct dorsal wall that is a part of the oviduct valve (See Figures 1a, b, and 2, Stage 7, in [90], marked by an asterisk).
**Oviducts, lateral**
FlyBase ID: FBbt:00004912 (lateral oviduct)
Definition: Epithelial tubes that connect the ovary to the common oviduct. They are lined with a chitinous intima, and surrounded by muscle. There are two lateral oviducts (one per ovary) in *D. melanogaster*. They usually have a loop near the base of the ovary in unmated females; the loop relaxes after mating to allow egg transit. [34,64].
**Oviduct valve**
FlyBase ID: FBbt:00052067
FlyBase synonyms: New term
Definition: The opening of the common oviduct into the uterine posterodorsal pouch. It consists of the oviduct valve flap and the oviduct dorsal ridge [90].
**Oviduct valve flap**
FlyBase ID: FBbt:00052069
FlyBase synonyms: New term
Definition: A chitinous flap that presses against the oviduct dorsal ridge, potentially blocking passage of substances between the uterus and the oviduct (See Figures 1a, b, and Figure 2, Stage 6 and Stage 7, in [90]).
**Oviprovector**
FlyBase ID: FBbt:00004926
FlyBase synonyms: Revised term. Previously used FlyBase synonyms include oviprotector [sic] (FBbt:00004831), *obsoleted;* vulva (FBbt:00004926)
Definition: Eversible membrane between the hypogynial valves that surrounds the vagina and the vulva [45,46,84].
Synonyms: dented membrane [35], vagina [95], vulva [24,34,35,68,73,74].
**Oviprovector dorsal membrane**
FlyBase ID: FBbt:00052070
FlyBase synonyms: New term
Definition: Dorsal membrane of the oviprovector connecting the hypogynial valves dorsally and surrounding the vulva dorsally. It bears no scales.
Synonyms: dorsal vulva [24,73].
**Oviprovector ventral membrane**
Flybase ID: FBbt:00052071
FlyBase synonyms: New term
Definition: Ventral membrane of the oviprovector connecting the hypogynial valves ventrally and the vulva laterally and ventrally. It bears oviprovector scales in serrated rows.
Synonyms: ventral vulva [24,73].
**Perineal membrane**
FlyBase ID: FBbt:00052072 (perineum)
FlyBase synonyms: New term
Definition: Intersegmental membrane connecting the female genitalia with the female analia. It extends from the posterior margin of tergite 7 to the ventral margin of the hypoproct. It is the place of type B copulatory wounds caused by the male epandrial posterior lobes during copulation.
Synonyms: female abdominal tergites 8/9 intersegmental membrane [31,71], female abdominal tergites 7/8 intersegmental membrane [30], perineal plate [46].
**Seminal receptacle**
FlyBase ID: FBbt:00004922
Definition: A compactly coiled epithelial tube connected to the anterior end of the uterus. The tube is long (1.7-2.7 mm) and slender, consisting of a proximal duct around 22 µm wide with a narrow lumen (2.5 - 4.5 µm) and a wider distal half (around 28 µm) with a larger lumen (12-19 µm). It is lined with a thin chitinous intima and surrounded by a nucleated sheath. The coil as a whole is surrounded by a sparsely nucleated peritoneal envelope. After copulation, the lumen of this tube is filled with spermatozoa. Sperm are stored in this structure after mating (Heifetz and Rivlin, 2010).
Synonyms: tubular receptacle [97], ventral receptacle [45,46,49,77,80,98–110].
**Spermatheca**
FlyBase ID: FBbt:00004921 (spermathecum)
Definition: A mushroom-shaped organ consisting of a capsule (end apparatus) connected to the uterus by a slender duct. Sperm is stored in this organ after mating [24,39,40,73,75,77–80,96,107]. The capsule is an inverted, double-walled bowl (i.e. outer capsule and basal introvert) consisting of sclerotized intima [62,111]. The intima is secreted by a layer of cuboidal epithelium, called the spermathecal secretory cells, which cover the outer wall [76]. After copulation, the lumen of the capsule is filled with spermatozoa. *D. melanogaster*typically has two spermathecae, but there is variation in spermatheca number within Drosophilidae [112].
**Spermathecal duct**
FlyBase ID: FBbt:00004923
Definition: Duct connecting the capsule of the spermatheca to the uterus. It opens into the uterine posterodorsal pouch, just posterior to the opening of the oviduct and close to the openings of the female accessory gland ducts.
**Spermathecal fat body**
FlyBase ID: FBbt:00052073 (spermathecal fat mass)
FlyBase synonyms: New term
Definition: A small and delicate mass of fat body that surrounds each of the spermatheca and is in close contact with the rectum [81].
**Spermathecal secretory cell**
FlyBase ID: FBbt:00052075
FlyBase synonyms: New term
Definition: Cell type that makes up the spermathecal secretory epithelium. Secretes the sclerotized intima of the spermathecal capsule [67,76].
Synonyms: gland cells [113].
**Spermathecal secretory epithelium**
FlyBase ID: FBbt:00052074
FlyBase synonyms: New term
Definition: A layer of cuboidal epithelium that surrounding the spermathecal capsule and secretes the sclerotized intima of the capsule [67,76].
Synonyms: gland cells [113].
**Uterus**
FlyBase ID: FBbt:00004924
FlyBase synonyms: Revised term
Definition: Anterior part of the genital chamber. It is an ectodermal invagination that is the site of egg fertilization. It is connected to the common oviduct anterodorsally and the vagina posteriorly. It is surrounded by muscles [24,90].
Synonyms: Bursa [56].
**Uterine anterior projection**
FlyBase ID: FBbt:00052076
FlyBase synonyms: New term
Definition: Inner elevation of the anterior wall of the uterus separating the uterine anterodorsal pouch and the uterine anteroventral pouch.
Synonyms: anterior uterus projection (See Figure 1bin 90).
**Uterine anterodorsal pouch**
FlyBase ID: FBbt:00052077
FlyBase synonyms: New term
Definition: Anterodorsal invagination of the uterus holding the openings of the common oviduct, the spermathecal ducts and the female accessory gland ducts. It is separated from the uterine dome by the uterine anterior projection.
Synonyms: pre-oviduct space (See Figures 1b, and 2, Stage 7 and Stage 10, in [90]).
**Uterine anteroventral pouch**
FlyBase ID: FBbt:00052078
FlyBase synonyms: New term
Definition: Anteroventral invagination of the uterus holding the opening of the seminal receptacle. It is separated from the uterine anterodorsal pouch by the uterine anterior projection.
Synonyms: vaginal pouch [103], uterine dome (See Figures 1b and 2, Stage 7, in [90]).
**Uterine furca**
FlyBase ID: FBbt:00052079
FlyBase synonyms: New term
Definition: The dorsal wall of the anterior part of the genital chamber (Figures 6d).
**Uterine furcal papillate elevation**
FlyBase ID: FBbt:00052080
FlyBase synonyms: New term
Definition: A thickened tissue of the uterine furca that forms the dorsal wall of the uterine anterodorsal pouch.
Synonyms: papillate elevation [76,90].
**Uterine posteroventral intima**
FlyBase ID: FBbt:00052081
FlyBase synonyms: New term
Definition: The ductal region between the base of the uterus and the exterior. The intima is thin in this region.
Synonyms: specialized vaginal intima [76,90], (Figure 1a, b).
**Vagina**
FlyBase ID: FBbt:00052061
FlyBase synonyms: Revised term
Definition: Posterior part of the genital chamber. It is an ectodermal invagination where insemination takes place. It extends from the posterior edge of the uterine specialized intima anteriorly to the vulva posteriorly [24,90].
**Vaginal furca**
FlyBase ID: FBbt:00052082
FlyBase synonyms: New term
Definition: The dorsal wall of the posterior part of the genital chamber.
**Vaginal furcal fold**
FlyBase ID: FBbt:00052083
FlyBase synonyms: New term
Definition: Folds in the inner membrane of the vaginal furca that can be recognized when the furca is extruded during oviposition (Figure 6a-c). It consists of a dorsal long fold, a pair of dorsolateral folds and a pair of lateral folds, which contact during copulation the male aedeagus, dorsal postgonites and ventral postgonites, respectively. The dorsolateral folds are the place of type A copulatory wounds [30].
**Vaginal furcal dorsal fold**
FlyBase ID: FBbt:00052084
FlyBase synonyms: New term
Definition: Dorsal fold of the vaginal furca that contacts the male aedeagus during copulation (Figure 6a).
Synonyms: uterine shield [15], vaginal shield [59].
**Vaginal furcal dorsolateral fold**
FlyBase ID: FBbt:00052085
FlyBase synonyms: New term
Definition: Dorsolateral fold of the vaginal furca that contacts the male dorsal postgonite during copulation (Figure 6b). It is the site of type A copulatory wounds [21,23]. There are two of these.
Synonyms: lateral folds [30], membranous pouch [59].
**Vaginal furcal lateral fold**
FlyBase ID: FBbt:00052086
FlyBase synonyms: New term
Definition: Lateral fold of the vaginal furca (Figure 6c). There are two of these.
**Vulva**
FlyBase ID: FBbt:00052087
FlyBase synonyms: Revised term
Definition: External opening of the vagina located medially between the posterior apices of the hypogynial valves. It is dorso-laterally surrounded by the oviprovector dorsal membrane and ventrally by the oviprovector ventral membrane. It is the copulatory orifice and site of exit for eggs.
Synonyms: genital opening [56], secondary gonopore [56].
**Setation**
**Epigynial sensilla**
FlyBase ID: FBbt:00110706
FlyBase synonyms: female abdominal tergite 8 bristle.
Definition: Small unpigmented sensilla on the ventral margin of the epigynial ventral lobe. There are 4 to 5 of these on each side.
**Epiproctal sensillum**
FlyBase ID: FBbt:00052088 (epiproctal bristle)
FlyBase synonyms: New term.
Definition: Any sensillum on the epiproct. There are on average 18 of these sensilla, of which two are large.
**Hypogynial sensilla**
FlyBase ID: FBbt:00052089 (hypogynial bristle)
FlyBase synonyms: New term
Definition: Any bristle on the outer or inner surface of a hypogynial valve. They are divided into 11-16 peg-like hypogynial teeth or thorns on the outer surface of a hypogynial valve, and 3 short and one long hypogynial trichoid sensilla on the inner surface of the hypogynial posterior lobe on each side.
Synonyms: vaginal teeth [52,114], ovisensilla [6]
**Hypogynial short sensillum**
FlyBase ID: FBbt:00004468
FlyBase synonyms: gonopod sensillum trichodeum
Definition: One of three minute apical sensilla on the inner surface of each hypogynial posterior lobe.
Synonyms: microbristle [39], sensillum trichodeum [24,35,70,73], ovisensillum trichoideum [72,84], apical ovisensillum trichoideum [49], inner ovisensillum [46], trichoid sensillum [7].
**Hypogynial long sensillum**
FlyBase ID: FBbt:00004467
FlyBase synonyms: gonopod long bristle
Definition: One long sub-apical sensillum on the inner surface of each hypogynial posterior lobe, ventral to the three hypogynial short sensilla.
Synonyms: long bristle [24,35,39,70,73,75], subapical bristle [40,80], subterminal hair [77], subapical ovisensillum trichoideum [49], inner ovisensillum [46], subapical sensillum [6], chaetic sensillum [7], subterminal inner ovisensilla [86], subterminal trichoid seta [115].
**Hypogynial tooth**
FlyBase ID: FBbt:00004466
FlyBase synonyms: gonopod thorn bristle
Definition: Any peg-like tooth on the outer surface of a hypogynial valve. There are 11-17 teeth of these on each side (Natascha Turetzek, personal communication).
Synonyms: peg-like bristle [75], tooth [40,43,77,80], spine bristle [35,39], thorn bristle [24,70,73], peg ovisensillum [84], peg [7,49], peg-like ovisensillum [72], outer ovisensillum [46], conical peg [7], hairlike sensillum [6], peg-like sensillum [6], peg sensillum [6], small proximal bristle [116], large distal bristle [116], vaginal tooth [52].
**Hypoproctal sensillum**
FlyBase ID: FBbt:00052090 (hypoproctal bristle)
FlyBase synonyms: New term
Definition: Any sensillum on the hypoproct. There are on average 19 of these sensilla, of which four are large.
**Oviprovector scale**
FlyBase ID: FBbt:00052091
FlyBase synonyms: New term
Definition: Scale-like projections on the surface of the ventral oviprovector membrane. These structures may act as ratchets to prevent bidirectional movement of an egg [32].
Definition: Muscle of the adult female abdominal segment 7 that extends to the uterine posteroventral intima [76].
**Musculature**
**Abdominal 7 female sternal muscle 144**
FlyBase ID: FBbt:00003510
Definition: Muscle of the adult female abdominal segment 8 that extends posteriorly along the epigynium to the epiproct. It is the most dorsal of the abdominal 8 female dorsal muscles [76].
**Abdominal 8 female dorsal muscle 145**
FlyBase ID: FBbt:00003469
Definition: Muscle of the adult female abdominal segment 8 that extends posteriorly along the epigynium to the epiproct. It is the most dorsal of the abdominal 8 female dorsal muscles [76].
**Abdominal 8 female dorsal muscle 146**
FlyBase ID: FBbt:00003470
Definition: Muscle of the adult female abdominal segment 8 that extends posteriorly from the epigynium to the hypoproct. It is ventral to the female dorsal muscle 147 [76].
**Abdominal 8 female dorsal muscle 147**
FlyBase ID: FBbt:00003471
Definition: Muscle of the adult female abdominal segment 8 that extends posteriorly from the epigynium to the uterus. It is dorsal to the female dorsal muscle 146 [76].
**Abdominal 8 female dorsal muscle 148**
FlyBase ID: FBbt:00003472
Definition: Muscle of the adult female abdominal segment 8 that extends dorsoventrally from the epigynium to the hypogynium. It is posterior to the female dorsal muscle 147 [76].
**Abdominal 8 female ventral muscle 149**
FlyBase ID: FBbt:00003486
Definition: Muscle of the adult female abdominal segment 8 that extends ventrally from the hypogynium to the uterus [76].
**Abdominal 8 female lateral ventral muscle 150**
FlyBase ID: FBbt:00003511
Definition: Muscle of the adult female abdominal segment 8 that extends along the lateral wall of the hypogynium from anterior to posterior [76].
**Abdominal 8 female transverse ventral muscle 151**
FlyBase ID: FBbt:00003512
Definition: Muscle of the adult female abdominal segment 8 that extends dorsoposteriorly from the hypogynium [76].
**Common oviduct circular muscle**
FlyBase ID: FBbt:00003553
Definition: A striated array of circular muscle fibres forming an almost continuous sheet around the common oviduct [76].
**Lateral oviduct circular muscle**
FlyBase ID: FBbt:00007338
Definition: A striated array of circular muscle fibres forming an almost continuous sheet around the lateral oviduct [76].
**Uterine circular muscle**
FlyBase ID: FBbt:00003554
Definition: Circular muscle that surrounds the adult female uterus [76].

Parenthetical names next to FlyBase ID numbers indicate the name as it appears in FlyBase, as some names were modified slightly in FlyBase for consistency with existing terms (e.g. “hypogynial bristle” is used as opposed to “hypogynial sensilla”, to reflect how other structures of this type have been referred to in FlyBase).

In a previous paper, we delineated the structures of the male terminalia of *D. melanogaster* and proposed a standard set of terms for these parts [22]. Following a discussion with the members of the consortium, we opted to designate terms that can be homologized across the Diptera, but we recommended that authors also indicate common terms, whenever possible, in their manuscripts in order to guarantee maximum understanding among disciplines. In this paper, we follow the same approach and collectively propose a collection of terms and definitions to unify the terminology associated with the female terminalia in *D. melanogaster* (Table 1). In contrast to our previous paper, which was limited to the external male terminalia, we also include here a comprehensive overview of the internal reproductive structures of females. Many of these structures make contact with intromittent parts of the male genitalia during mating and may therefore be of interest in studies of genital evolution and coevolution [e.g. 15, 21, 23].

Distinguishing between the various parts of the female genitalia can be challenging, especially where clear boundaries (e.g. sutures, joints) do not exist. To achieve maximum clarity in our visual depictions, we have used a combination of bright field images of dissected cuticle (Canton S wild type strain), scanning electron microscopy, and line drawings.

## Results and discussion

### *A visual atlas of adult* D. melanogaster *female terminalia*

We provide below a unified nomenclature of the anatomical parts of the female terminalia of *D. melanogaster*, together with images to visualize the various structures with as much clarity as possible. We hope that this effort will facilitate the study of female terminalia in *D. melanogaster* and related species by providing both a common language for cross-reference and delimitations for features of interest.

The female terminalia of *D. melanogaster* are composed of anatomical structures arising from a fusion of abdominal primordia 8–10 [24,25]. In females, the abdominal segment 8 primordium of the genital disc develops into the majority of the internal and external female genital structures [26,27]. The abdominal segment 9 primordium is reduced in females, giving rise to the internal structure of the female accessory gland and the dorsal surface of the uterus [26,28]. In females, as in males, the abdominal segment 10 primordium of the genital disc develops to become the analia [29]. We divide our descriptions of the terminalia into two regions, internal and external. The external terminalia have prominent roles in oviposition and copulation, while the internal terminalia have roles in ovulation and sperm storage. We use the junction of the oviprovector (external) and vulva (internal) as the division between these two regions.

Table 1 details our proposed unified nomenclature. Each proposed term is listed along with a description of the structure, previously used alternate names, and references. For ease of conversion, Table 2 provides the reverse search functionality; previously used terms are listed in the first column, with the corresponding unified nomenclature term we propose here given in the second column. Instances where the same term has been used elsewhere for more than one distinct structure are indicated with an asterisk.

**Table 2.** Table of correspondence between terms previously used in publications and term of the standardized nomenclature

Previous terminology	Synonym in the new nomenclature	Reference[s)
abdominal sternite 9	hypoproct	42
abdominal tergite 10	epiproct	56
abdominal tergite 9	epiproct	42
anterior uterus projection	uterine anterior projection	90
apical ovisensillum trichodeum	hypogynial short sensillum	49
appendicular gland	female accessory gland	56
basal isthmus	hypogynial anteroventral bridge	77, 80
bursa	uterus	56
chaetic sensillum	hypogynial long sensillum	7
colleterial gland	female accessory gland	56
conical peg	hypogynial tooth	7
dented membrane	oviprovector	35
distal part of the oviscapt valve	hypogynial posterior lobe	46
dorsal anal plate	epiproct	69, 70, 74, 88
dorsal part of the female abdominal tergite 8	epigynial dorsal lobe	69
dorsal vaginal plate	hypogynial posterior lobe	69, 70, 74, 88
dorsal vulva	oviprovector dorsal membrane	24, 73
dorsodistal depression	hypogynial posterodorsal pouch	46
egg guide	hypogynium	77, 80
female abdominal sternite 8	hypogynium	56
female abdominal tergite 8	epigynium	24, 42, 45, 48, 68–70
female abdominal tergite 9	epigynium	31, 71
female abdominal tergites 7/8 intersegmental membrane	perineal membrane	30
female abdominal tergites 8/9 intersegmental membrane	perineal membrane	31, 71
female abdominal tergite 8 bristle	epigynial sensillum	FlyBase term
female gonopod	hypogynium	42
female proctiger	female analia	31, 42
female sternite 9	furca	56
genital fork	furca	56
genital opening	vulva	56
gonopod long bristle	hypogynial long sensillum	FlyBase
gonopod sensillum trichodeum	hypogynial short sensillum	FlyBase
gonopod thorn bristle	hypogynial tooth	FlyBase
gland cells	spermathecal secretory cells	113
hairlike sensillum	hypogynial tooth	6
heavily sclerotized bar	hypogynial anteroventral bridge	42
inner ovisensillum*	hypogynial long sensillum	46
inner ovisensillum*	hypogynial short sensillum	46
large distal bristle	hypogynial tooth	116
lateral folds	vaginal furcal dorsolateral fold	30
long bristle	hypogynial long sensillum	24, 35, 39, 70, 73, 75
lower anal plate	hypoproct	40
lower margin of the egg-guide lobe	hypogynial anterior lobe	80
membranous pouch	vaginal furcal dorsolateral fold	59
microbristle	hypogynial short sensillum	39
outer ovisensillum	hypogynial tooth	46
ovipositor	hypogynium	35, 40, 48, 75
ovipositor scales	oviprovector scales	32
oviprotector	oviprovector	FlyBase
oviscape	hypogynium	30, 31, 47, 84
oviscapt	hypogynium	15, 30, 45–47,72, 49, 85, 86
oviscapt pouch	hypogynial posterodorsal pouch	15
ovisensillum	hypogynial sensillum	6
ovisensillum trichodeum	hypogynial short sensillum	72, 84
papillate elevation	uterine furcal papillate elevation	76, 90
parovarium	female accessory gland	24, 56, 73, 77–80
peg	hypogynial tooth	7, 49
peg ovisensillum	hypogynial tooth	84
peg sensillum	hypogynial tooth	6
peg-like bristle	hypogynial tooth	75
peg-like ovisensillum	hypogynial tooth	72
peg-like sensillum	hypogynial tooth	6
perineal plate	perineal membrane	46
pre-oviduct space	uterine anterodorsal pouch	90
secondary gonopore	vulva	56
sensillum trichodeum	hypogynial short sensillum	24, 35, 70, 73
small proximal bristle	hypogynial tooth	116
specialized vaginal intima	uterine posteroventral intima	76, 90
spermathecum	spermatheca	FlyBase
spine bristle	hypogynial tooth	35, 39
subanal plate	hypoproct	76
subapical bristle	hypogynial long sensillum	40, 80
subapical ovisensillum trichoideum	hypogynial long sensillum	49
subapical sensillum	hypogynial long sensillum	6
submedian incision of the egg-guide lobe	hypogynial mid-dorsal incision	80
subterminal hair	hypogynial long sensillum	77
subterminal inner ovisensilla	hypogynial long sensillum	86
subterminal trichoid seta	hypogynial long sensillum	115
supraanal plate	epiproct	76
thorn bristle	hypogynial tooth	24, 70, 73
tooth	hypogynial tooth	40, 43, 77, 80
trichoid sensillum	hypogynial short sensillum	7
tubular receptacle	seminal receptacle	97
upper anal plate	epiproct	40
upper margin of the egg-guide lobe	hypogynial posterior lobe	80
uterine dome	uterine anteroventral pouch	90
uterine shield	vaginal furcal dorsal fold	15
uterus	genital chamber	39, 73, 79
vagina*	genital chamber	FlyBase
vagina*	oviprovector	95
vaginal apodeme	furca	56
vaginal plates	hypogynium	24, 39, 68, 73, 74, 79, 87
vaginal pouch	uterine anteroventral pouch	103
vaginal sheild	vaginal furcal dorsal fold	59
vaginal teeth	hypogynial sensillum	52, 114
vaginal tooth	hypogynial tooth	52
ventral anal plate	hypoproct	24, 68, 70, 73–75
ventral part of the female abdominal tergite 8	epigynial ventral lobe	69
ventral receptacle	seminal receptacle	45, 46, 49, 77, 80, 98–110
ventral vaginal plate	hypogynial anterior lobe	69, 70, 74, 88
ventral vulva	oviprovector ventral membrane	24, 73
vulva	oviprovector	35

* Note that these previously used terms correspond to multiple anatomical parts in the new nomenclature

### External structures of the female terminalia

**Figure 3.**
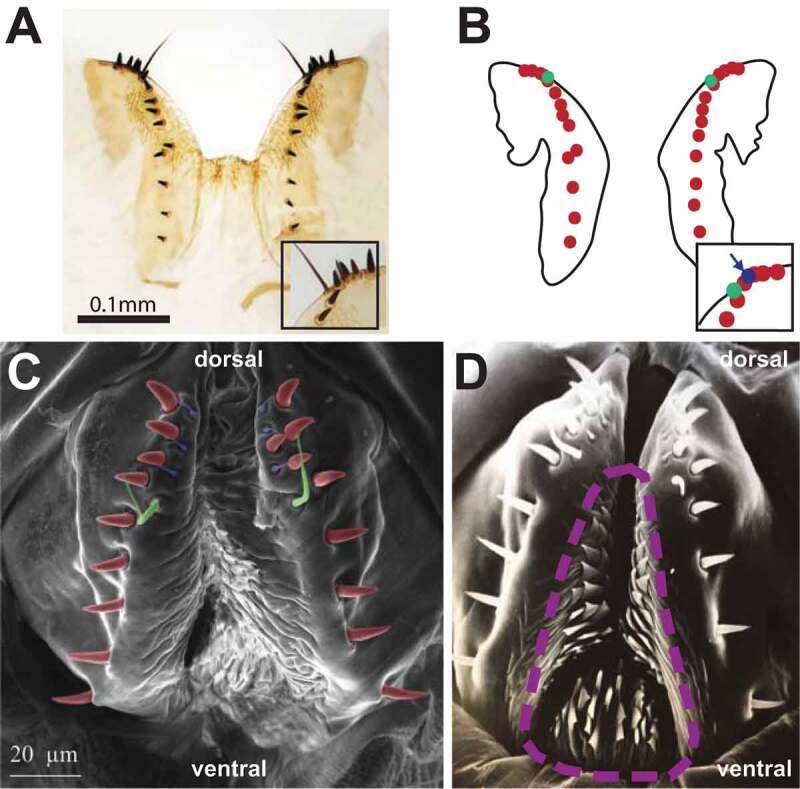
**Hypogynial sensilla. [A]** Light microscopy image of hypogynial lobes. Inset is a closeup of the posterior tip of one lobe. **[B]** Line tracing of **[A]**, showing locations of bristle types. Hypogynial short sensilla are barely visible from this angle, but one is shown in the inset (arrow). **[C]** Scanning electron micrograph of female genitalia, posterior view. Colour-coding of sensilla types is as follows: Red, hypogynial tooth; Green, hypogynial long sensillum; Blue, hypogynial short sensillum. **[D]** Scanning electron micrograph of female genitalia, posterior view. The region covered with oviprovector scales is indicated with a dashed purple line.

The external structures of the female terminalia (Figures 1–3) consist of the female analia and external genitalia, both of which harbour sensilla (bristles). In females, the analia (Figure 2, **panel A”**) are subdivided into a dorsal plate (the epiproct), and a ventral plate (the hypoproct). The analia are surrounded by the genital tissue of the epigynium (formerly female abdominal tergite 8). The epigynial ventral lobe connects to the paired valves (left and right) of the hypogynium via the perineal membrane. We further subdivide the hypogynium into several parts (Figure 2, **panel A’**). The hypogynial posterior lobe and hypogynial anterior lobe are the posterior and anterior parts of each valve of the hypogynium. The ventral side of both valves is connected by the hypogynial anteroventral bridge (Figure 2, **panel B’**). The hypogynial mid-dorsal incision is an indentation on the outside of each hypogynial valve. The posterior and anterior hypogynial lobes are delimited by an imaginary line connecting the hypogynial mid-dorsal incision with the hypogynial long sensillum. During copulation, the male surstylus contacts the hypogynium near this incision [30]. The hypogynial posterodorsal pouch is a depression positioned at the apical end of each hypogynial valve (Figure 2, **panel A’**), which contacts the male epandrial posterior lobe early in copulation [15,31]. The two hypogynial valves are connected medially by the oviprovector, an eversible membrane whose ventral surface bears the oviprovector scales (Figure 2, **panel C**; Figure 3d), which likely act to prevent bidirectional movement of eggs [32].

The setation of the external female terminalia has several readily identifiable components (Figure 3). Sensilla on the epiproct and the hypoproct are referred to as epiproctal sensilla and hypoproctal sensilla, respectively (Figure 2, **panel A**). On the genitalia, both the epigynium (epigynial sensilla) and hypogynium (hypogynial sensilla) have characteristic setation. The hypogynial sensilla are subdivided into three types (Figure 3). Hypogynial short sensilla (previously gonopod sensillum trichodeum; Figure 3c, blue) are small apical bristles at the dorsal tip of the hypogynial posterior lobe. The hypogynial posterior lobe of each valve also possesses a single hypogynial long sensillum (previously gonopod long bristle; Figure 3b,c, green) at the apical end. Finally, each valve of the hypogynium possesses a row of stout hypogynial teeth (previously gonopod thorn bristles or vaginal teeth; Figure 3b,c, red).

### Internal female genital and reproductive structures

**Figure 4.**
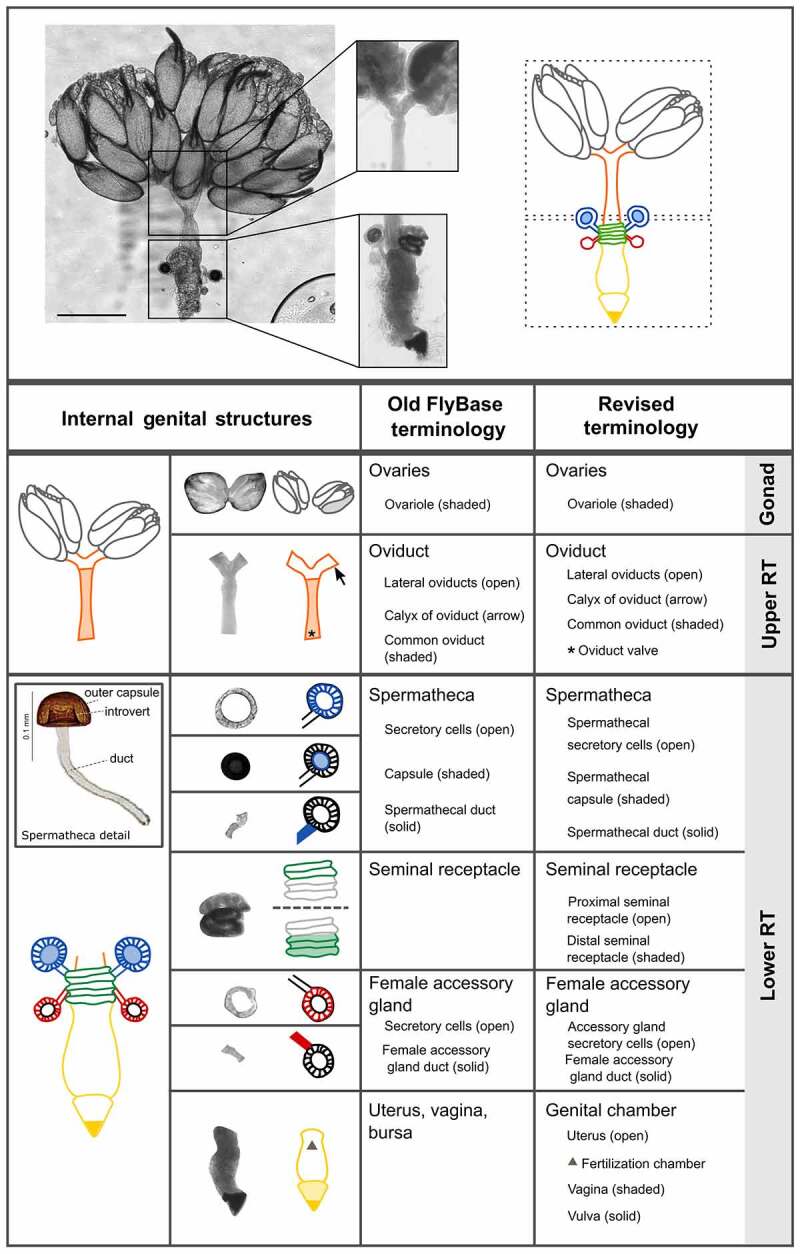
**Visual atlas of internal female genitalia and reproductive structures**. Confocal bright-field images and schematic of *Drosophila melanogaster* female (Canton S strain) reproductive system. Scale bar is 500 µm. The upper box shows the upper reproductive tract (upper RT) and the ovaries, the lower box is the lower reproductive tract (Lower RT). The lower panel displays individual structures with line drawings to aid identification. The internal structures and substructures include the gonad (ovaries), the upper RT (oviduct) and the lower RT (seminal receptacle, spermatheca, female accessory glands, genital chamber). Inset is a detail of the spermatheca to highlight substructures. Previous FlyBase terms are on the left and revised terms are on the right.

**Figure 5.**
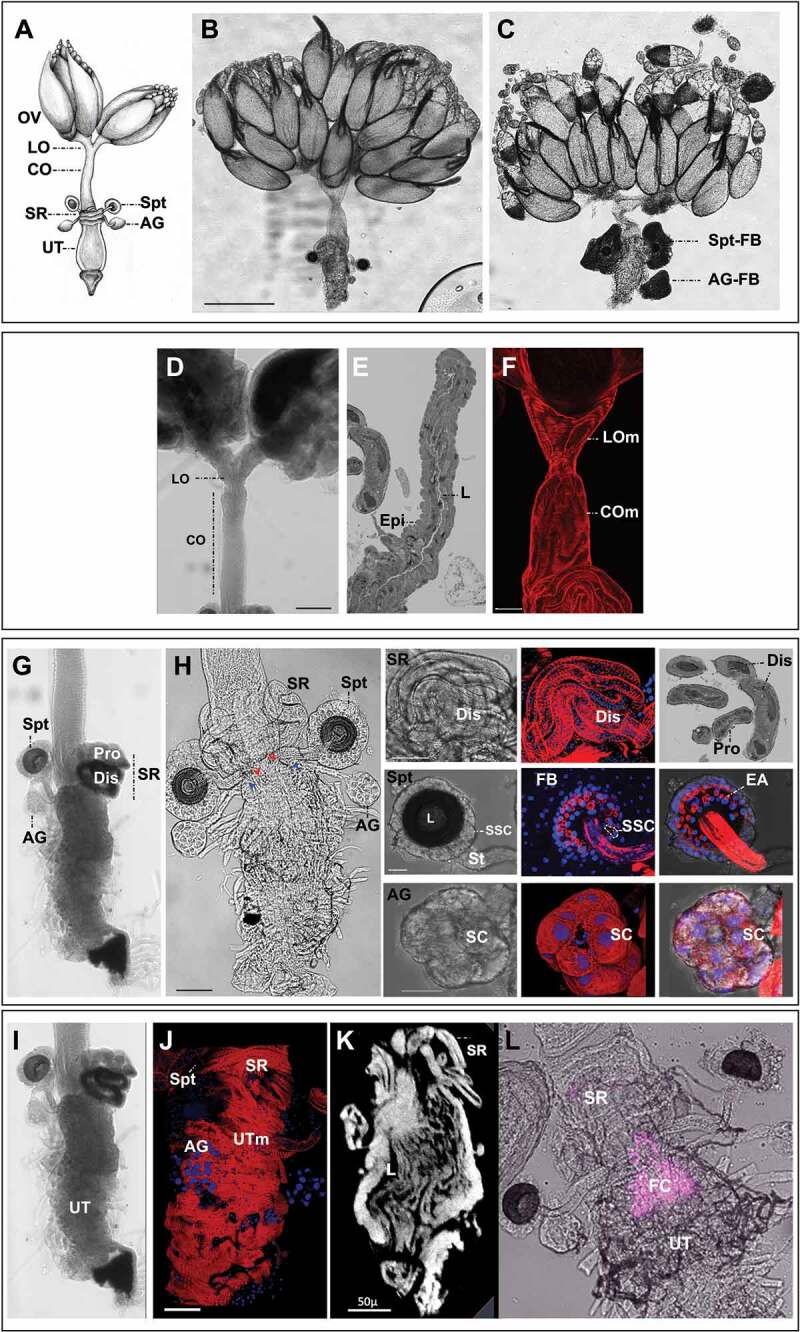
**Internal genital structures of the female reproductive tract. [A]** The *Drosophila* female reproductive tract consists of a pair of ovaries (OV) connected to a median common oviduct (CO) by two lateral oviducts (LO), and a uterus (UT) that leads to the vagina, which opens to the exterior through the vulva. The reproductive tract also includes specialized organs: a pair of spermathecae (Spt), seminal receptacle (SR), and a pair of female accessory glands (AG); drawing by Zohar Nir-Amitin. **[B, C]** The whole system with fat body **[C]** or without the fat body **[B]** that covers the spermatheca (Spt-FB) and the female accessory glands (AG-FB) (scale bar is 500 µm). **[D-F]** Upper RT that includes the lateral and common oviducts (scale bar is 100 µm), **[D]**. Toluidine blue stained 1 µm thick section of the oviduct that highlight the luminal space (l) and the epithelial cells (Epi) **[E]**. Upper RT stained with Alexa Fluor 594-phalloidin (red) showing the muscle fibres in different regions of oviduct (scale bar is 50 µm), **[F]. [G, H]** Lower reproductive tract, including the spermatheca (Spt), seminal receptacle (SR), and female accessory glands (AG). Note the red and blue arrowheads that mark the connection of the Spt and AG stalks to the uterus (scale bar is 50 µm). The panel also presents bright-field, phalloidin and DAPI images: SR showing the proximal (Pro) and distal (Dis) regions and the surrounding layer of visceral muscle (scale bar is 50 µm); Spt showing the spermathecal secretory cells (SSC), the lumen where sperm is stored (L), the stalks (St) (scale bar is 20 µm), the end apparatus (EA), and the fat body (FB, stained with DAPI) that surrounds the Spt; the female accessory glands (AG) showing the secretory cells (SC) (scale bar is 20 µm). **[I-L]** Zoom-in image of the uterus: **[J]** layers of circular muscle fibres (UTm) (scale bar is 50 µm), **[K]** micro-CT of the uterus highlighting the structure of the uterine lumen (L) (scale bar is 50 µm), **[L]** DsRed expression (magenta) showing the location of the fertilization chamber (FC), a structure to which the stalks of the SR, Spt and AG enter.

The upper reproductive tract consists of the ovaries and oviducts, which transfer mature eggs to the lower reproductive tract (Figures 4, 5). The lower reproductive tract is composed of the genital chamber, female accessory glands, seminal receptacle and spermathecae. The seminal receptacle and spermathecae store sperm after mating, while the female accessory glands and the spermathecal secretory glandular cells that surround the spermatheca capsule serve as secretory organs. The genital chamber is subdivided into the uterus (or bursa; anterior) and vagina (posterior) (Figure 4). It is in the uterus that fertilization of eggs takes place [33]. The posterior opening of the lower reproductive tract consists of the vagina through which sperm is transferred to the female and the vulva, a name which has also previously been used for the oviprovector, and where copulation occurs and where the egg exits the reproductive tract [e.g. 34, 35].

### Delineation of structures

Some parts of the female genitalia that we outline in this work do not have clear boundaries, such as ridges or clefts. We justify the demarcation of these structures in several ways. In some cases, we note the structure separately because the feature appears to have functional significance. For instance, the hypogynial mid-dorsal incision (Figure 2) does not have clear boundaries with surrounding tissue, but there is evidence to suggest that this depression is a site that makes contact with the male surstylus during copulation [30]. Delimitation of anatomical features can also be aided by considering the distribution of important developmental molecules (e.g. transcription factors), the patterning of which may indicate regions that harbour developmental or evolutionary independence [27,28,36]. Lastly, some identified features are quite subtle in *D. melanogaster* but are more exaggerated in closely related species, providing reasoning for their designation as notable structures of the female genitalia in this group. For example, the hypogynial posterodorsal pouch is relatively shallow in *D. melanogaster* but is unambiguous in *D. simulans* [15], a closely related species which diverged about 2 million years ago [37]. Future work investigating the development and function of these structures will further aid in structural demarcation.

### Choice of terms

The term hypogynium was first proposed by Crampton [38] to refer to the abdominal sternite below the genital apparatus of the female, which in the case of Diptera corresponds to sternite 8. In the same paper, Crampton [38] defined the term hypandrium as the abdominal sternite below the genital apparatus of the male, *i.e*. sternite 9 in Diptera. Whereas the term hypandrium has been used in *Drosophila* systematics and developmental biology as early as the 1940s [e.g. 39, 40], ‘hypogynium’ has never been applied to *Drosophila*. Instead, a variety of non-anatomical terms such as ‘egg-guide’ and ‘ovipositor’ have been applied to the female egg-laying external structures. In entomology, the ovipositor is usually formed from the appendices of the genital segment [41], and indeed Ferris [42] called the external egg-laying structure (in *D. melanogaster*) the ‘female gonopod’. However, it has been suggested that Diptera females lack an ovipositor, in the proper entomological sense [41]. Indeed, in *D. melanogaster* the homoeotic gene *Abdominal-B* represses all leg-development genes in female A8, confirming the sternal nature of the hypogynium [43]. Crampton [44] suggested that specific terms, such as oviscapt, would be more appropriate. Grimaldi [45] has introduced this term in *Drosophila* systematics, and since then it has been used in multiple systematic and functional morphology studies [15,30,46,47,72]. However, given our conservation of the terms hypandrium and epandrium for the sternite and tergite of abdominal segment 9 in our paper on male terminalia anatomy [22], we prefer here for consistency the usage of the terms hypogynium and epigynium for the sternite and tergite of female abdominal segment 8. As the anatomical term hypogynium is not commonly used in the literature, it would be preferable to cite it alongside the more common functional term ‘ovipositor’ in publications, e.g. hypogynium (ovipositor) or ovipositor (hypogynium).

The analia have formerly been called the proctiger and consequently the sternite and tergite surrounding the anus were called the hypoproct and the epiproct, respectively [30,45,46,72,48,49]. However, in some Dipteran species, two additional lateral plates, called the cerci, also surround the anus. Remarkably, there are no hypo- and epiprocts in males and no cerci in females of *D. melanogaster*. Nonetheless, it has been observed that in *doublesex, transformer-2, hermaphrodite*, or *intersex* mutant females, the hypoproct is reduced and the epiproct shifts laterally, resembling the male cerci, but still usually fused on the dorsal side [50–53]. This suggests that the female epiproct may have the same developmental origin as both male cerci. Females of the subfamily Steganinae have a pair of lateral plates identified as cerci posterior to the epiproct [45]. In the subfamily Drosophilinae, however, these cerci have been lost or possibly fused to the epiproct. In addition, we note that in some insect groups (such as odonates) the terms hypoproct, epiproct, and paraproct describe terminal structures that are not functionally homologous to the structures named here for *D. melanogaster* and could very well derive from different segment primordia during terminalia development [54,55].

**Figure 6.**
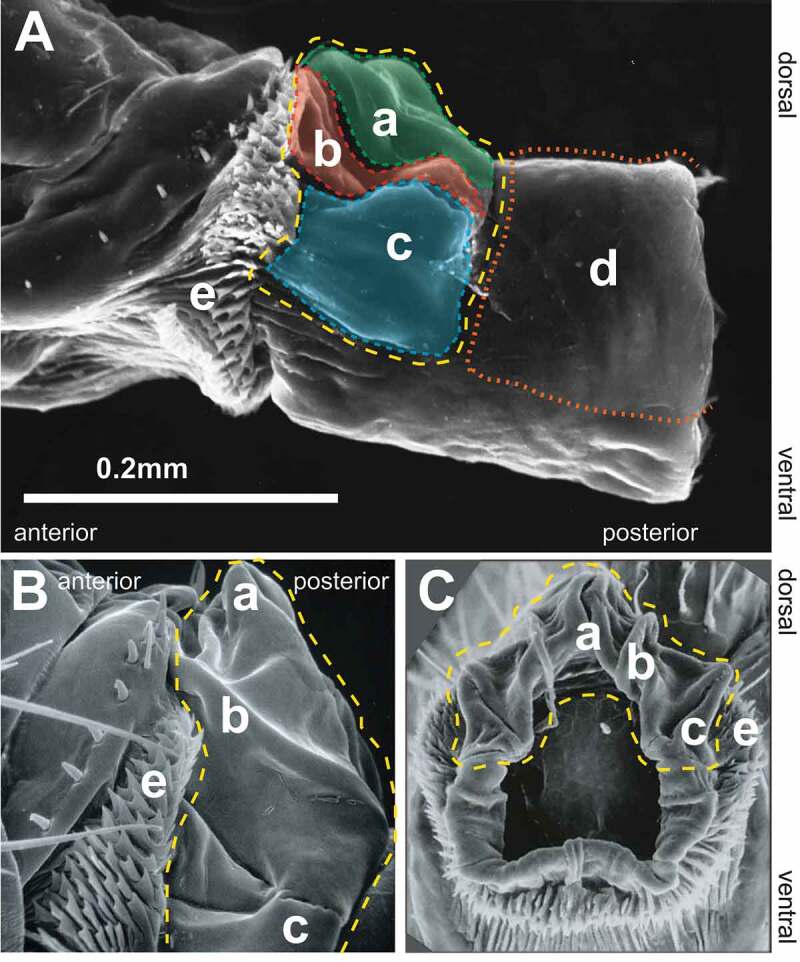
Scanning electron micrographs of the furca and furcal folds. **[A]** Lateral view with internal structures extruded, **[B]** lateral view, unextended, **[C]** posterior view. In each image, the vaginal furca is indicated by the yellow dashed line. **a**. vaginal furcal dorsal fold, **b**. vaginal furcal dorsolateral fold, **c**. vaginal furcal lateral fold, **d**. uterine furca. Not shown in the figure is the portion of the uterine furca that extends internally until the entry point of the spermathecal and accessory gland ducts into the genital chamber. **e**. oviprovector scales.

Considering the internal structures, we propose here a term in *Drosophila*, the furca (Figure 6). In non-Cyclorrhaphan Diptera, the furca is an internalized sclerite on the dorsal surface of the genital chamber derived from sternite 9 [56], and it was believed to be absent or unrecognizable in most Cyclorrhapha. Interestingly, developmental studies showed that the dorsal wall of the genital chamber in *D. melanogaster* derives from the A9 primordium [26], suggesting the furca is present in this species though far less sclerotized. The furca has several folds that we choose to define more precisely here, motivated by evidence that some of these may interact with male intromittent organs. For example, the vaginal furcal dorsolateral fold (Figure 6b) is the location of one type of copulatory wound described by Kamimura and Mitsumoto [30].

### Incorporation of our standardized terminology across areas of research and species

A primary goal of this article is to facilitate the flow of information across disciplines and research areas. To this aim, we worked with the FlyBase team to incorporate our unified terminology into their database, updating and adding terms as needed. We understand that there may be good reasons for individual authors to continue using the terminology that they are accustomed to in their own work. In such cases, our suggestion would be to parenthetically reference the unified terminology that we outline here, e.g. parovaria (female accessory glands). In this manner, there will be greater ease in translating across works that employ different terminology for the same feature.

Our work here focused on the terminalia of *Drosophila melanogaster* females. However, despite the great morphological diversity of the female genitalia in the Drosophilidae, the general ground plan of these structures is fairly well conserved. Therefore, most of the terms we define are easily extensible to other species, facilitating comparison across studies outside *D. melanogaster*. In cases where structures have been lost in *D. melanogaster* (and thus are not named here), we hope that this set of unified terms will mitigate potential confusion by giving common references for surrounding structures. We briefly illustrate below two exciting research areas for which our unified terminology may prove useful in facilitating fruitful comparisons across species or in different species groups.

#### Evolution of genitalia in response to ecological factors

Evolution of the female genitalia has frequently taken place in response to changing oviposition substrates. Adaptations usually involve changes in size and shape of the hypogynium as well as in the number, disposition and shape of the hypogynial sensilla [6,16]. For example, species laying eggs on solid substrates, such as *D. suzukii* or leaf- and bark-breeding Hawaiian drosophilids [57], often have large and elongate hypogynium with numerous, large teeth-like sensilla. On the other hand, species laying eggs in decaying or soft tissues, such as *D. melanogaster*, often have a short and roundish hypogynium with fewer and less sharp sensilla. In some cases, female genital evolution in *Drosophila* has important consequences for agriculture. For example, the evolution of a serrated ovipositor in *D. suzukii* and closely related species (e.g. *D. subpulchrella*) has allowed these flies to oviposit in ripening fruit, making them crop-damaging pests, while closely related species such as *D. biarmipes*, where the ovipositor retains the basal shape and setation, are benign [16]. A common language with respect to anatomical structures will ensure that studies conducted in disparate systems come together to inform our collective understanding of the forces and mechanisms driving such changes in response to ecological factors.

#### Coevolution of the sexes

The rapid evolution of animal genitalia is a longstanding area of research interest [1–3]. While early work focused specifically on male structures, added emphasis has recently been placed on understanding the evolution of female structures [5,10], and how coevolution of male and female genitalia might contribute to the rapid evolution of these structures in both sexes [11,12]. Adaptations of male genitalia to rapidly evolving female genitalia [58], or vice versa, usually involve changes concerning specific genital features, such as the shape and size of the hypogynial posterodorsal pouch in the *melanogaster* species complex and the sclerification of some internal walls of the oviprovector (e.g. in *D. teissieri*), the vulva (e.g. in *D. orena*) and the vagina (e.g. in *D. erecta*) [15,59,60]. Some internal sperm-storage organs, such as the seminal receptacle, have co-evolved with the size of the male sperm [61,62]. We hope that the common set of terms we outline here to reference the various parts of the female genitalia, in combination with the previous work outlining terms for the male genital structure [22], will aid in the synthesis of empirical studies of genital evolution and coevolution across Drosophilid species.

## Methods

### Scanning electron microscopy

The scanning electron micrographs from Figures 1b and 3d were collected about 40 years ago, and the exact strain of *D. melanogaster*, and exact methods used to collect these images are no longer known.

The scanning electron micrograph in Figure 3c was collected as follows: Adult female *D. melanogaster* were fixed in 2% glutaraldehyde in 0.1 M sodium cacodylate, stained with osmium tetroxide, dried through an ethanol series (35–100%) and the ethanol dried with a Tousimis AutoSamdri 815 critical point dryer. The terminalia were then dissected from the abdomen, mounted on stubs, and coated with gold-palladium using a Tousimis sputter coater. Specimens were visualized with a Hitachi SM-5000 scanning electron microscope.

The scanning electron micrograph in Figure 6a was collected by fixing a female sample from the Oregon-R strain in ice-cold ethanol, followed by a t-butanol wash, and drying by sublimation. The samples were then gold-coated and observed under a scanning electron microscope (Hitachi S-510).

The scanning electron micrographs in Figure 6b, c, are from L. Tsacas’ collection at the National Museum of Natural History, Paris (Courtesy of the Museum).

### Bright field cuticle imaging

For cuticle images in Figure 2 **(except** Figure 2**, panel C)**, a Canton S line of *Drosophila melanogaster* (Bloomington # 64349) was used. Adult females were dissected in 100% EtOH with micro-forceps and mounted in PVA Mounting Medium (BioQuip). Samples were imaged at 10× and 20× magnification on a Leica DM 2000 with a Leica DFC540 C camera. Images were Z stack-compiled with the Leica Application Suite to allow for optimal focus.

For cuticle image in Figure 2c and the image of the spermatheca in Figure 4 **(inset**), female specimens from a lab-culture strain of Canton S were used. The distal portion of abdomen after the segment 7 including the spermatheca therein was detached from the main body in 70% EtOH, treated with 10% KOH solution at 80–90°C for about 5 min, and mounted in a droplet of glycerine on a cavity slide. After removing the tergite and sternite 7 within glycerine, the dissected and cleaned terminalia and spermatheca were microphotographed at different depths of focus using a DinoLite® Digital Eyepiece Camera attached to an Olympus BX50 microscope. The photos were stacked into an all-in-focus composite using the software CombineZP [63]. The confocal images were edited using Adobe Photoshop CS6 and Adobe Illustrator CS6.

For the cuticle image in Figure 3a, a female from the Canton S strain was used. The sample was mounted in 50:50 Hoyer’s medium and lactic acid. The sample was imaged at 20× magnification using a Zeiss Axioplan with a Manta G609C camera (Allied Vision Technologies). Focus stacking was performed with the software Picolay (www.picolay.de, version 2020–08-13).

### Visualization of the upper and lower RT

Reproductive tracts were dissected in Schneider’s Drosophila medium (Sigma) on ice and processed for electron microscopy as described in 64. Briefly, tracts were flat-embedded between two sheets of Aclar (Electron Microscopy Sciences), which allowed us to image the entire tract at the light microscopic level prior to sectioning. Sections were cut on a Reichert Ultracut microtome. One-µm thick sections were stained with 1% toluidine blue and viewed with a Zeiss Axioplan microscope.

### Immunocytochemistry

Reproductive tracts were dissected in Yamamoto’s Ringer, fixed in 4% paraphormaldehyde in PBS and incubated in blocking solution and stain with Alexa Fluor 594-phalloidin (1:200) and DAPI (Molecular Probes) as described in 64.

Reproductive tracts of the different treatments were mounted with Antifade media [65**]** on a multi-well glass slide.

### Reporter constructs

The image in Figure 5L shows the pattern of *DsRed* expression (magenta) for an enhancer-reporter construct containing 301 bp of sequence between the transcription start site of *CG32833* and a distal transcription start site of *twist* (coordinates 2,2985,299–2,2985,599 in *D. melanogaster* genome v6.42). Note that this intergenic sequence is also upstream of the transcription start site of *miR-4939* (transcribed in the same direction as *CG32833*) and of the transcription start site of long non-coding RNA gene *CR42742* (transcribed in the same direction as *twist*). It is not known to which gene’s expression pattern the reporter corresponds. The 301-bp fragment was amplified by PCR with primers respectively containing a KpnI site and an XhoI site, for cloning into the KpnI and XhoI sites in the polylinker of pRed H-Stinger [66]. The construct was inserted into strain *w*^1118^ by *P*-element-mediated transformation, and the reproductive tract of a female from the resulting strain was dissected and imaged as done previously [67].

### Confocal microscopy

Reproductive tracts were imaged in a Leica TCS SP8 multiphoton (MP) laser scanning confocal microscope operated by the LAS X software. Fluorescence was detected by using argon excitation lasers of 488 nm captured by a conventional photomultiplier (PMT). Image processing was done using Fiji and Imaris 8.4 (Bitplane).

### Micro computed tomography (micro-CT)

Reproductive tracts were stained with a mixed contrasting dye [1% crystalline I2 (Merck 376,558) and 1% Tannic acid (Merck 1401–55-4) in 200 proof ethanol] for 24–48 hours at 40°C. Before imaging, the samples were washed two times for ten minutes each in fresh 200 proof ethanol. Micro-CT was done with a Zeiss Xradia micro XCT-400 at X20 magnification and data processing was done using AVIzO and Fiji (Zelinger E, Brumfeld V, Rechav K, Heifetz Y, in prep).

## Data Availability

Data sharing is not applicable to this article as no new data were created or analyzed in this study.
